# Mendelian Randomization Integrating GWAS, eQTL, and mQTL Data Identified Genes Pleiotropically Associated With Atrial Fibrillation

**DOI:** 10.3389/fcvm.2021.745757

**Published:** 2021-12-17

**Authors:** Yaozhong Liu, Biao Li, Yingxu Ma, Yunying Huang, Feifan Ouyang, Qiming Liu

**Affiliations:** ^1^Department of Cardiovascular Medicine, Second Xiangya Hospital, Central South University, Changsha, China; ^2^Department of Cardiology, Asklepios Klinik St. Georg, Hamburg, Germany

**Keywords:** atrial fibrillation, Mendelian randomization, multi-omics, GWAS, eQTL, mQTL

## Abstract

**Background:** Atrial fibrillation (AF) is the most common arrhythmia. Genome-wide association studies (GWAS) have identified more than 100 loci associated with AF, but the underlying biological interpretation remains largely unknown. The goal of this study is to identify gene expression and DNA methylation (DNAm) that are pleiotropically or potentially causally associated with AF, and to integrate results from transcriptome and methylome.

**Methods:** We used the summary data-based Mendelian randomization (SMR) to integrate GWAS with expression quantitative trait loci (eQTL) studies and methylation quantitative trait loci (mQTL) studies. The HEIDI (heterogeneity in dependent instruments) test was introduced to test against the null hypothesis that there is a single causal variant underlying the association.

**Results:** We prioritized 22 genes by eQTL analysis and 50 genes by mQTL analysis that passed the SMR & HEIDI test. Among them, 6 genes were overlapped. By incorporating consistent SMR associations between DNAm and AF, between gene expression and AF, and between DNAm and gene expression, we identified several mediation models at which a genetic variant exerted an effect on AF by altering the DNAm level, which regulated the expression level of a functional gene. One example was the genetic variant-cg18693985-CPEB4-AF axis.

**Conclusion:** In conclusion, our integrative analysis identified multiple genes and DNAm sites that had potentially causal effects on AF. We also pinpointed plausible mechanisms in which the effect of a genetic variant on AF was mediated by genetic regulation of transcription through DNAm. Further experimental validation is necessary to translate the identified genes and possible mechanisms into clinical practice.

## Introduction

Atrial fibrillation (AF) is the most common sustained arrhythmia. The current global prevalence of AF is between 2 and 4%, and a 2.3-fold rise is expected (1). Well-known risk factors for AF include advanced age, male sex, obesity, smoking, and cardiovascular comorbidities (2). AF increases the risk of stroke, dementia, and depression; and contributes to a 1.5~3.5 fold increase in mortality (1). Despite its epidemiological importance, the fundamental mechanisms that underlie AF remain poorly understood.

It is estimated that the impact of common genetic variants on the risk of AF is ≈ 22% (3). Large-scale genome-wide association studies (GWAS) have identified more than a hundred loci associated with AF (4, 5). However, the genes or DNA regulatory elements through which these variants exert their effects on AF are still unclear. The summary data-based Mendelian randomization (SMR) was developed to integrate GWAS data with molecular traits data, such as cis-expression quantitative trait loci (cis-eQTL) study and cis-DNA methylation QTL (cis-mQTL) study. This method has been successfully applied to prioritize gene expression or DNA methylation (DNAm) sites that are pleiotropically or potentially causally associated with complex traits (6, 7).

**Figure 1:**
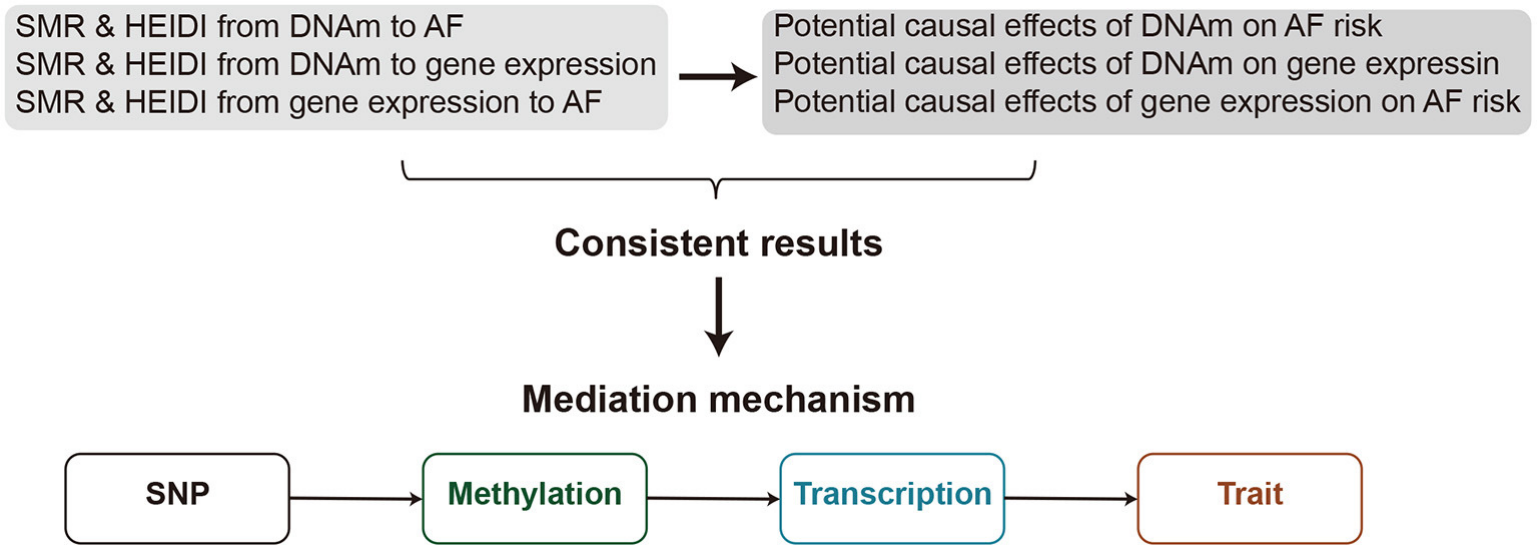
A flowchat to identify mediation mechanism. The effects of DNAm on trait, DNAm on gene expression, and gene expression on trait are evaluated using the SMR & HEIDI method and integrated to identify potential mediation mechanisms in which an SNP exerts an effect on the trait by altering the DNAm level, which then regulates the expression levels of a functional gene.

Most of the AF-associated variants resided in non-coding regions of the genome (4, 5). Therefore, it is rational to assume that these variants affect AF risk by regulating the transcriptional output. In the present retrospective study, we adopted the SMR approach to prioritize functional genes or DNAm sites for AF by leveraging the largest AF GWAS summary statistics and large-scale blood cis-eQTL/cis-mQTL data. Further, by incorporating consistent pleiotropic associations between DNAm and AF, between gene expression and AF, and between DNAm and gene expression, we detected plausible mediation models for AF susceptibility genes in which the effect of a genetic variant on AF was mediated by genetic regulation of transcription through DNAm (Figure 1). These results provide important leads for the design of future studies to understand the functional mechanism by which DNA mutations lead to AF and to develop new therapeutic targets.

## Materials and Methods

### Data Sources for Integrative Analysis

This study was approved by the Institutional Committee on Human Research at the Second Xiangya Hospital. The genetic association data for AF was obtained from the largest GWAS study (4), which included 60,620 cases and 970,216 controls of European ancestry. The eQTL summary-level statistics were obtained from the CAGE study (8), which consisted of 2,765 individuals (predominantly Europeans) with gene expression measured at the transcription level in peripheral blood. The mQTL data was a meta-analysis (7) of blood mQTL from the Brisbane Systems Genetics Study (*n* = 614) and the Lothian Birth Cohorts of 1921 and 1936 (*n* = 1,366). All the individuals were of European ancestry. We used the annotation file from Price et al. (9) to annotate the closest genes of DNA methylation probes. For eQTL and mQTL, only probes having at least one cis-QTL passing the genome-wide significant threshold (P-QTL < 5 × 10^−8^) were included in the SMR analyses. The cis region was defined as within 2 Mb of a probe in either direction.

### SMR and HEIDI for Detecting Pleiotropic Association

The SMR test was introduced to test the association between the exposure and the outcome using a single genetic variant, usually the single nucleotide polymorphism (SNP), as the instrumental variable (6). Detailed methods have been described elsewhere (6). Briefly, let y be the outcome, x be the exposure, and z be the instrumental variable. The Mendelian Randomization (MR) estimate of the effect of exposure on the outcome (b_xy_) is the ratio of the estimated effect of the instrument on the outcome (b_zy_) to that on the exposure (b_zx_). When testing for a DNAm/transcript–trait association, x, y, and z refer to DNAm/transcript, trait, and the top-associated cis-mQTL/cis-eQTL, respectively. When testing for a DNAm–transcript association, x, y, and z refer to DNAm, transcript, and the top-associated cis-mQTL, respectively. Bear in mind that SMR based on a single genetic variant is unable to distinguish between causality and pleiotropy. Therefore, the pleiotropic association identified should only be interpreted as an inference about causality. The heterogeneity in dependent instruments (HEIDI) test was done to test against the null hypothesis that there is a single causal variant underlying the association. We adopted the default settings in SMR (The window centered around the probe to select cis-eQTL/mQTL was set as 2 Mb; *p*-value threshold to select the top associated eQTL/mQTL for the SMR test was set as 5.0 × 10^−8^; the threshold of QTL *p*-value to select QTLs for the HEIDI test was set as 1.57 × 10^−3^, which is equivalent to a chi-squared value of 10; and removing SNPs in very strong linkage disequilibrium with the top associated QTL). For SMR & HEIDI test between DNAm and transcript, we only focused on the analysis in the cis-region: only testing for associations between DNAm sites and gene expression probes that were in <2 Mb distance. For the SMR test, we used Bonferroni correction to account for multiple testing.

### Functional Enrichment Analysis and Tissue-Specific Expression Analysis

To functionally annotate the identified genes, we conducted gene ontology (GO) enrichment analysis using the “ClusterProfiler” package (10) in R software.

We also conducted tissue-specific enrichment analysis (TSEA) using the “deTS” package (11) implemented in R. The Genotype-Tissue Expression (GTEx) project reference panel was applied. For each tissue, the top 5% genes with the highest t-statistics were predefined as significantly enriched in the tissue. The overlap between AF-related genes and the genes enriched in each tissue was evaluated by Fisher exact test. The Bonferroni correction was used to adjust for multiple testing, and the significance cutoff was set as *P* < 0.05/47 = 0.001 because 47 tissue groups were tested.

### SMR Analysis for AF With EQTL Data From Other Heart-Related Tissue

In the main analyses, we used eQTL and mQTL data from the blood samples as a proxy to heart tissue to maximize power for discovery. To test whether eQTL from blood could well-represent those from AF/heart-related tissues, we further performed the SMR analysis using eQTL data sets of right atrial appendage, left ventricle, and skeletal muscle from the GTEx Consortium.

## Results

### SMR to Prioritize Candidate Gene Targets and DNAm Sites

**Figure 2:**
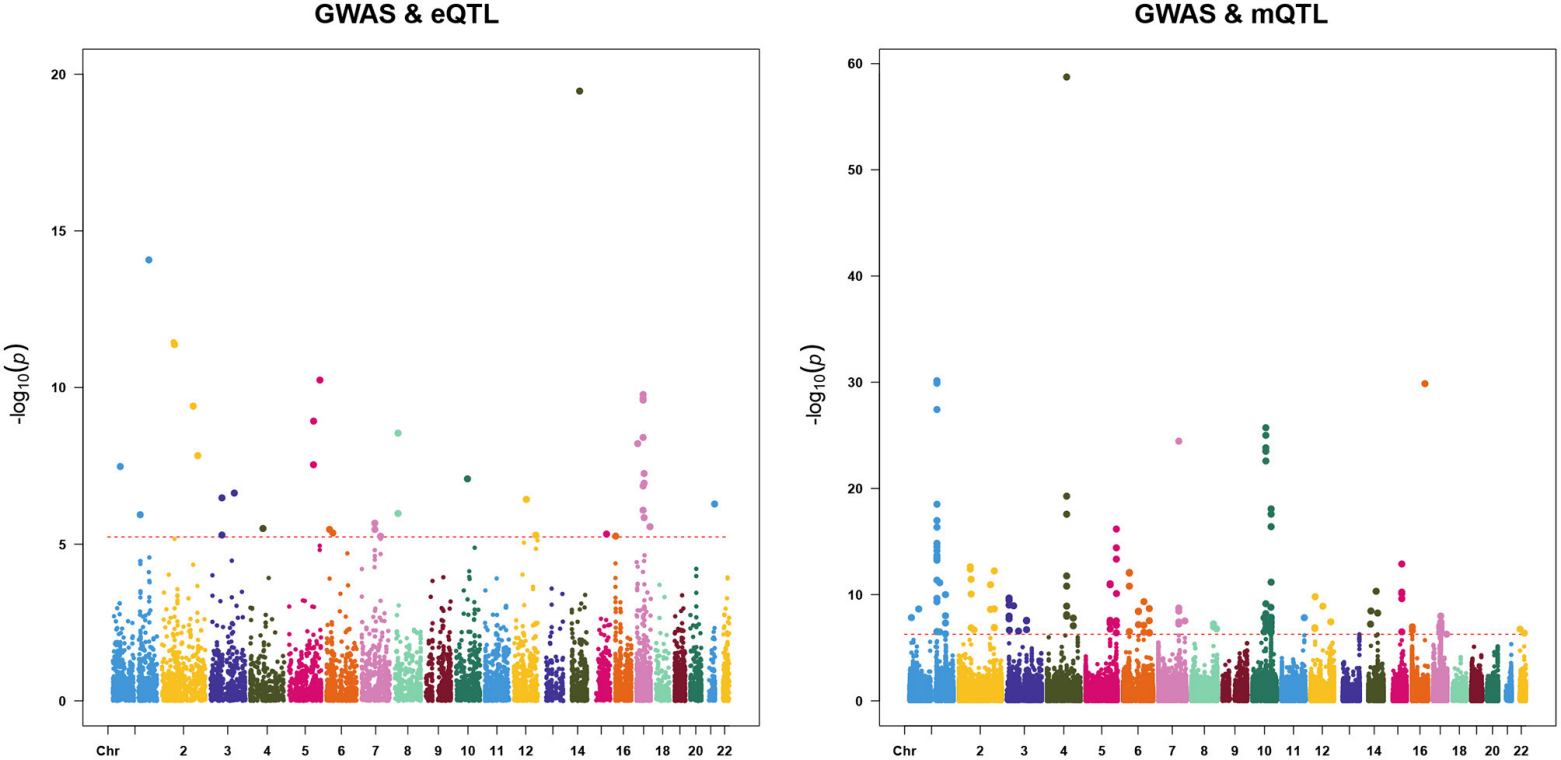
Result of summary data-based Mendelian randomization (SMR). Shown are –log10 (*P*-values) from the SMR tests for AF against the physical positions of gene expression (left) or DNAm probes (right). Red dot lines represent the significance thresholds of the SMR tests.

We first used the SMR & HEIDI method to identify pleiotropic associations between gene expression and AF and between DNAm and AF. We only used genetic instruments in the cis region to minimize the possibility of pleiotropy while maximizing the possibility of causality. The results of SMR analysis in two omics-level were plotted in Figure 2. Using the cis-eQTL summary data from the CAGE study, we identified 39 transcript probes (corresponding to 36 genes) with false discovery rate (FDR) <0.05 (equivalent to *P* < 0.05/8507). After the HEIDI test, 23 probes (corresponding to 22 genes) were not rejected at a threshold of 0.01 (probes that could not be used to conduct the HEIDI test were also excluded). In the analysis with DNAm data, we identified 167 DNAm probes (corresponding to 88 closest genes) associated with AF with FDR <0.05 (equivalent to *P* < 0.05/93211), of which 80 DNAm probes (corresponding to 50 closest genes) passed the HEIDI test. By integrating results from transcriptome and methylome, 66 genes were finally pinpointed and 6 of them existed in both two omics levels (Supplementary Tables 1, 2).

GO annotation (BP, biological process; CC, cellular component; MF, molecular function) enrichment analyses were conducted to clarify the biological functions of the 66 identified genes. The enriched GO annotations included muscle system process (GO:0003012) and actin filament-based movement (GO:0030048) in the BP category; cell leading edge (GO:0031252), axolemma (GO:0030673), neuron spine (GO:0044309), and voltage-gated potassium channel complex (GO:0008076) in the CC category; phosphatidylinositol bisphosphate binding (GO:1902936), superoxide-generating NAD(P)H oxidase activity (GO:0016175), voltage-gated potassium channel activity (GO:0005249), and actin-binding (GO:0003779) in the MF category. TSEA analysis showed the identified genes were significantly enriched in right atrial appendage (*P* = 3.6 × 10^−4^) and left ventricle (*P* = 6.4 × 10^−3^) after multiple testing and were enriched in skeletal muscle at a threshold of *P* = 0.05.

### Identifying Consistent SMR Associations Across Multi-Omics

Having identified pleiotropic associations between DNAm and AF, and between gene expression and AF, we then aimed to integrate the two omics results. To do this, we explored possible associations between the 80 DNAm and the 23 gene expression probes (DNAm as the exposure and transcript as the outcome). Thirty-five DNAm probes were detected to show significant pleiotropic associations with 12 gene expression probes at FDR <0.05, of which 13 pairs passed the HEIDI test (corresponding to 10 DNAm and 8 gene probes). These pairs could be explained by the median model (Figure 1) that the effects of genetic variants in the AF loci are mediated by the regulation of gene expression through DNAm (Supplementary Table 3).

**Figure 3:**
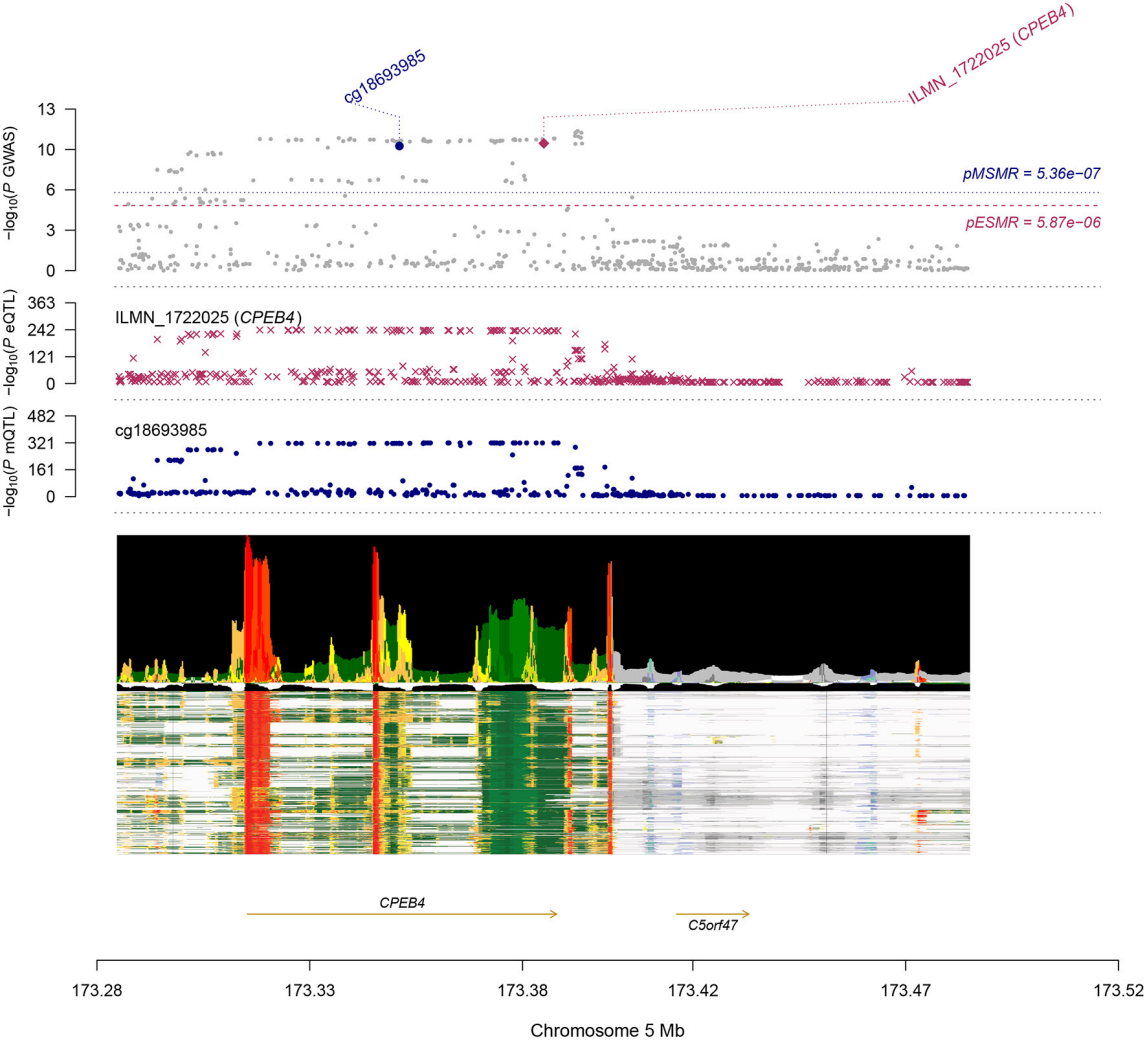
Results of SNP and SMR associations across mQTL, eQTL, and GWAS at the CPEB4 locus. The top plot shows –log10 (*P*-values) of SNPs from the AF GWAS. The red diamond and blue circle represent –log10 (*P*-values) for probes from the SMR tests for associations of gene expression and DNAm probes, respectively. The second plot shows eQTL results for the probe ILMN_1722025 (tagging CPEB4). The third plot shows mQTL results for the DNAm probe cg18693985. The bottom plot shows 18 chromatin state annotations (indicated by colors) of 833 samples from EpiMap for different primary cells and tissue types (rows).

Most of the DNAm sites in DNAm-transcript associations were located in promoter and enhancer regions of the associated genes, as shown in Wu's study (7). These offer opportunities to infer the genetic regulatory mechanism at a GWAS locus. A notable example is the cg18693985-CPEB4-AF axis (Figure 3). The b-SMR of DNAm to AF, DNAm to gene, and gene to AF was 0.044, −0.897, −0.049, respectively. Indeed, the DNAm probe (cg18693985) resides in the enhancer region of CPEB4 (ILMN_1722025) across multiple cells and tissues, as annotated from the Epigenome Integration Across Multiple Annotation Projects (EpiMap) (12). Furthermore, the top associated SNP in the mQTL & GWAS analysis of the cg18693985 is only 14 kbp away from the top associated SNPs in the eQTL & GWAS analysis of ILMN_1722025. CPEB4, also called the cytoplasmic polyadenylation element-binding protein 4, is an RNA-binding protein. It was recently identified as a critical regulator of cardiomyocyte function (13). Depletion of CPEB4 led to pathological cellular growth both *in vitro* and *in vivo* in cardiomyocytes and decreased cardiac function (13). Taken together, we hypothesize a mechanism in which a genetic variant at the enhancer region of CPEB4 gene led to DNAm, which down-regulated the expression of the CPEB4 gene (for instance, by disrupting the binding of a transcription factor). Decreased expression of CPEB4 then increased the risk of AF.

### Tissue Specificity and Robustness

To examine whether blood eQTL data are good representatives of eQTL from other AF/heart-related tissues, we conducted the SMR analysis for AF using 3 eQTL data sets from the GTEx, including right atrial appendage, left ventricle, and skeletal muscle. Only 14 of the 22 genes were available in the SMR and HEIDI tests. Of the 14 genes, 10 genes (71.4%) passed both the SMR test (*P* < 0.05) and HEIDI test (*P* > 0.01) in at least one of the 3 eQTL data sets. The high replication rate verified the feasibility of using blood sample as a proxy to identify genes.

## Discussion

To our knowledge, this is the first study to integrate GWAS, eQTL, and mQTL data of AF. We used the SMR approach to explore putative genes and DNAm sites that showed pleiotropic or potentially causal associations with AF. Of the 66 identified genes, 26 of them were previously associated with AF at the genetic level in the GWAS catalog (https://www.ebi.ac.uk/gwas/) including CASZ1, CAV2, CDKN1A, CEP68, COG5, CPEB4, HCN4, IL6R, KCNH2, KDM1B, LRIG1, MAPT, NEURL, NKX2-5, PHLDB2, PITX2, PPFIA4, PTK2, RPL3L, RPS2, SOX5, SPATS2L, SSPN, SYNE2, SYNPO2L, and TNFSF13. Our study added their roles in AF at the epigenomic or transcriptomic level. For instance, an increased transcript expression level of interleukin 6 receptor (IL6R) was associated with increased AF risk in the eQTL & GWAS analysis (b_SMR = 0.184). This is consistent with the well-established role of the IL6/IL6R inflammatory signaling as a biomarker of cardiovascular events risk (14). Further, previous studies have found that the causal non-synonymous variant Asp358Ala in the IL6R locus was associated with decreased risks of AF (15) and postoperative AF (16). This variant was also associated with a decreased odds of coronary heart disease events (17), the effect of which was consistent with IL6R blockade from infusions of tocilizumab in patients with rheumatoid arthritis studied in randomized trials. We could therefore expect the use of IL6R antibodies in preventing AF risk. Another example is the PITX2 locus, which has been well-investigated in both population-based and experimental-based AF studies (18). From the mQTL & GWAS analysis, we identified 4 DNAm sites in the PITX2 locus to be associated with AF risk, and 3 of them resided in/near the enhancer region across heart-related tissues, as annotated from the EpiMap (12). The positive b_SMR values for the 3 DNAm implicated a novel mechanism that a causal genetic variant in the PITX2 locus affected AF risk by altering the DNAm level, which then regulated the gene expression.

For other 40 “new” genes, some of them have also been studied in AF by biological experiments such as ADORA1 and KCNIP2. The ADORA1 gene encodes for the adenosine a1 receptor. Adenosine is a well-known endogenous nucleoside and can provoke spontaneous or pacing-induced AF. Blockade of adenosine receptors could prevent AF occurrence (19). The KCNIP2 gene encodes for potassium voltage-gated channel interacting protein 2, which mediates the transient outward K^+^ current (Ito) in cardiac tissue. Decreased expression of KCNIP2 was observed in the right atrial appendage of AF patients (20) and the atrial tissue of transgenic mice model that was used to display spontaneous AF and action potential prolongation (21). In conclusion, the identified genes from the eQTL & GWAS and mQTL & GWAS analyses provided future directions for linking genetics with the disease phenotype and for developing new therapeutic targets.

Compare with other transcriptome-wide association methods, the SMR method allows us to incorporate different types of molecular traits. This is another innovation in our AF study. By combining the SMR results from transcriptome to AF, methylome to AF, and methylome to transcriptome, we identified several mechanical models at which a genetic variant exerted an effect on AF by altering the DNAm level, which then regulated expression of the downstream gene. One example is the cg18693985-CPEB4-AF axis. Even there was no direct experimental evidence, our studies provided a strong indication of the participation of CPEB4 in AF pathogenesis, as supported by the consistent SMR associations across DNAm, transcript, and GWAS. Notably, in a recent work, Riechert et al. reported CPEB4 as a key regulator of cardiac growth and function. CPEB4 knockdown increased the expression of NPPA–NPPB cluster (13), which are established risk markers for AF (22). Our results suggested CPEB4 as a potential drug target for AF prevention and treatment.

In their recent work, Wang et.al developed a strategy to integrate multiple omics data to identify AF-related genes (23). They integrated the AFGen 2017 GWAS result, a whole blood methylome-wide association study, and a whole blood transcriptome-wide association study, and identified nearly 2000 AF genes. Compare to their study, our study focused on identifying the regulatory mechanisms at a GWAS locus. The identified genes that passed the SMR and HEIDI test have a pleiotropic association with AF, that is, the gene expression (or the DNAm level) and the trait are affected by the same underlying causal variant. Even though this method could not diminish the possibility of pleiotropy, which describes a genetic variant that is associated with multiple traits, our study provides a list of prioritized genes and DNAm sites for follow-up functional studies that linking genetics with epigenome and transcriptome. Another advantage of our study is that we also conducted the SMR & HEIDI test between methylome and transcriptome. The consistent associations of DNAm with gene expression, of DNA with AF, and of gene expression with AF, reinforce our confidence in the functional relevance of the genes and provided possible mechanisms as illustrated in Figure 1.

We acknowledged several limitations. First, we used the blood eQTL and mQTL as a proxy. This is mainly because of the difficulty in obtaining heart tissue, and there are limited eQTL or mQTL data available for intuitively AF-related tissue, such as the left atrium and the pulmonary veins. However, the large sample size in blood data maximized the statistical power, and it has also been proved that blood could be used as a good proxy for other tissues such as brain (24). Moreover, most of the genes identified in the blood eQTL could be replicated in the eQTL data of GTEx heart tissue, which further reinforced the availability of identified genes. Second, we only focused on the analysis in the cis-region. The benefit of focusing on cis-region is to minimize the possibility of selecting pleiotropic variants so able to increase the potentiality of causality. However, this is at the cost of losing the discovery of genes or DNAm sites that showed trans- relationships. Third, all of our study samples were from participants of European ancestry. Future GWAS or eQTL/mQTL studies conducted in other populations, such as Asians, might help to enhance the generalizability of our findings. Forth, the benefit of adding DNAm data is at the cost of statistical power if we only focus on the associations passing the SMR & HEIDI test in all three steps. Further development of the method that integrates multi-omics data in a single test is a priority in the future. Finally, only eQTL and mQTL data were included in this study, future studies could incorporate more types of molecular traits, such as splicing QTLs (sQTLs) and protein QTLs (pQTLs).

In conclusion, our SMR analysis identified multiple genes and DNAm sites that showed pleiotropic or potentially causal associations with AF. We also pinpointed plausible mechanisms in which the effect of a genetic variant on AF was mediated by genetic regulation of transcription through DNAm. Further experimental validation is necessary to translate AF-related genes and the identified possible mechanisms into clinical practice.

## Data Availability Statement

The datasets presented in this study can be found in online repositories. The names of the repository/repositories and accession number(s) can be found in the article/Supplementary Material.

## Author Contributions

YL and BL performed the bioinformatic analysis and were the major contributors in writing the manuscript. YM and YH made important modifications to the manuscript. YL, FO, and QL designed the research project and created the final revision of the manuscript. All authors read and approved the final version of the manuscript.

## Funding

This work was supported by grants from the National Natural Science Foundation of China (Grant Nos. 81770337 and 82070356).

## Conflict of Interest

The authors declare that the research was conducted in the absence of any commercial or financial relationships that could be construed as a potential conflict of interest.

## Publisher's Note

All claims expressed in this article are solely those of the authors and do not necessarily represent those of their affiliated organizations, or those of the publisher, the editors and the reviewers. Any product that may be evaluated in this article, or claim that may be made by its manufacturer, is not guaranteed or endorsed by the publisher.
